# A new cell line based coculture system for skin sensitisation testing in one single assay using T cells, aryl hydrocarbon receptor knockout, and co-inhibitory blockage

**DOI:** 10.1007/s00204-023-03506-3

**Published:** 2023-05-05

**Authors:** Anna Sonnenburg, Ralf Stahlmann, Reinhold Kreutz, Matthias Peiser

**Affiliations:** 1 Institute for Clinical Pharmacology and Toxicology, Charité-Universitätsmedizin Berlin, Berlin, Germany; 2 Institute of Chemistry and Biochemistry, Freie Universität Berlin, Berlin, Germany; 3 Department Pesticides Safety, German Federal Institute for Risk Assessment, Berlin, Germany; 4 Department Food Safety, German Federal Institute for Risk Assessment, Berlin, Germany

**Keywords:** Skin sensitisation, New approach methodologies, T cell activation, AhR knockout, Anti-PD-L1, CRISPR/Cas

## Abstract

Established in vitro assays for regulatory testing of skin sensitisation partly suffer from only moderate sensitivity, specificity, and predictivity when testing specific groups of chemicals. This may be due to limited biomarker response in vitro in cell types that interact as crucial players of in vivo skin sensitisation pathogenesis. Here, we propose a molecular approach to overcome this limitation. In our model, we apply genome editing and blocking of immunoregulatory molecules to increase the range of biomarker modulation by sensitising chemicals. To this end, aryl hydrocarbon receptor (AhR) knockout was done by CRISPR/Cas9 technology in THP-1 cells and combined with Programmed Cell Death-Ligand (PD-L)1 blockade. AhR-knockout THP-1 in coculture with HaCaT keratinocytes showed increased CD54 expression compared to wild type cells after stimulation with 10 µmol/L dinitrochlorobenzene (DNCB) that was further enhanced by anti-PD-L1. After stimulation of AhR-knockout THP-1 with 200 µmol/L mercaptobenzothiazol or 10 µmol/L DNCB, cocultivated Jurkat T cells significantly increased expression of T cell receptor-associated CD3. No such increase was detected after prior treatment of THP-1 with 150 µmol/L of irritant sodium lauryl sulphate. Additionally, higher levels of inflammatory cytokines MIP-3α, MIP-1β, TNF-α, and IL-8 were found in supernatants of enhanced loose-fit co-culture based sensitisation assay (eLCSA) after substance treatment. Hence, eLCSA allowed to discriminate between sensitisers and non-sensitisers. Thus, inhibition of immunoinhibitory pathway signalling by combining AhR knockout and PD-L1 antibody blockage into an assay involving main acting cell types in skin sensitisation may increase sensitivity and specificity of such assays and allow potency derivation.

**Supplementary Information:**

The online version contains supplementary material available at 10.1007/s00204-023-03506-3.

## Introduction

Skin sensitisation and its clinical manifestation, allergic contact dermatitis (ACD), are common adverse reactions to distinct chemicals used in cosmetics, personal care, biocidal and plant protection products, and topical drugs. The OECD’s Adverse Outcome Pathway Knowledge Base project established an adverse outcome pathway (AOP) for skin sensitisation as the first adopted AOP in the project (OECD 2014). The first step in this AOP is a molecular initiating event (MIE, also sometimes referred to as key event 1) which comprises covalent binding of low molecular weight compounds to skin proteins. The further key events (KE) in this AOP are activation of keratinocytes (KE2), activation of dendritic cells (KE3), and activation and clonal expansion of antigen specific T cells (KE4). The adverse outcome is skin sensitisation as evidenced by manifestation of ACD after a second contact to the causative agent.

The European Union banned animal testing on the endpoint skin sensitisation for cosmetic product ingredients in 2013. Therefore, alternative methods had to be developed. There are now OECD validated methods available for the first three KE (OECD 2018a; OECD 2018b; OECD 2022). However, until now, there is no validated method for the last and crucial KE, the activation and clonal expansion of T cells. This is due to several reasons. First, the use of primary human T cells leads to extreme donor dependent biological variability (van Vliet et al. 2018). Second, activation of dendritic cells by chemicals in vitro is relatively weak and thus presumably fails to induce subsequent activation of T cells.

Immunosuppressive mechanisms, which act to prevent an exaggerated immune response upon dendritic cell activation, are thought to dampen the measurable response of cells in vitro. These inhibitory mechanisms are mediated through several specific receptors, like aryl hydrocarbon receptor (AhR) and programmed cell death 1 (PD-1) and its ligand (PD-L1). The AhR has a variety of functions within the immune system, which can be immunosuppressive as well as activating depending on involved cell types and other factors (Esser and Rannug 2015; Stockinger et al. 2014). Moreover, the AhR exhibits diverse functions within the cell cycle, in haematopoiesis, and prenatal development (Esser 2012; Hao and Whitelaw 2013).

The PD-1/PD-L1 system is a known target for cancer immunotherapy as it helps immune escape in certain tumour types (Prestipino and Zeiser 2019). It was also shown to be involved in the development T cell anergy in transplant tolerance (Baas et al. 2016) and in the regulation of antigen-presenting cell and T cell activity in skin allergy (Hitzler et al. 2012; Pena-Cruz et al. 2010).

Therefore, we have aimed to investigate whether inhibition of the function of these receptors would lead to an enhanced T cell response to allergen activated dendritic cells in vitro. The targets were inhibited by different methods. PD-L1 was blocked using a monoclonal antibody and AhR was knocked out by CRISPR/Cas9 technique. Thus, a targeted combination of the modifications was possible and allowed to evaluate potential synergistic effects. HaCaT keratinocytes, THP-1, and Jurkat T cells, all cells lines which are commonly employed as surrogates for primary human cells were used to avoid donor specific variability.

We have modified an in vitro sensitisation assay developed by our working group—the loose-fit co-culture based sensitisation assay (LCSA) that was originally established with primary human cells (Schreiner et al. 2007; Wanner et al. 2010). Primary keratinocytes were already switched to HaCaT in our previous work (Frombach et al. 2017). The LCSA was developed as co-culture of human keratinocytes and in situ generated dendritic cell-related cells and was then enhanced by introduction of a primary T cell response in later developments (Frombach et al. 2018).

Here, AhR-knockout (AhR-k.o.) and/or PD-L1 blocked THP-1 were co-cultured with HaCaT keratinocytes and treated with an extreme or a moderate allergen (2,4-dinitrochlorobenzene—DNCB or 2-mercaptobenzothiazole—MBT), or an irritating substance (sodium lauryl sulphate–SLS). That way activated THP-1 were then co-cultured with Jurkat T cells. Allergen-specific response was monitored by flow cytometric measurement of T cell proliferation and cell surface marker expression, and by ELISA of secreted cytokines. Thus, we here present a two-tiered test system (see supplemental figures for tier description) that provides measurable endpoints for dendritic cell and T cell activation, thereby KE3 and KE4 of AOP40 in one assay. Furthermore, the assay could potentially be used to cover keratinocyte activation (KE2) as well. The test system was named enhanced Loose-fit Coculture-based Sensitisation Assay (eLCSA).

Our results show that that eLCSA using AhR-knockout THP-1 combined with PD-L1 blockade enhances allergen-induced responses in cocultivated Jurkat T cells and thereby provides a complete and competent allergy testing system.

## Materials and methods

### Cells and chemicals

Cell lines THP-1 and Jurkat were purchased from the German Collection of Microorganisms and Cell Cultures (DMSZ, Braunschweig, Germany), HaCaT cells were used from a stock available at the institute. Cell culture conditions for proliferation are described elsewhere (Sonnenburg et al. 2023). All cells were kept in medium containing penicillin/streptomycin (10,000 U/mL/10 mg/mL, Merck) for the first several passages. Thereafter, cells were maintained in antibiotic free media.

Test chemicals (2,4-dinitrochlorobenzene [DNCB] 97%, CAS no. 97-00-7; 2-mercaptobenzothiazol [MBT] 97%, CAS no. 149–30-4; sodium lauryl sulphate [SLS] 92.5–100.5% based on total alkyl sulphate content, CAS no. 1335-72-4), dibutylphthalate (DBP, ≥ 98%, CAS no. 84-74-2), and 6-thioguanine (6-TG, ≥ 98%, CAS no. 154-42-7) were purchased from Sigma Aldrich (now Merck) and dissolved in DMSO or PBS (Sigma). FACS antibodies and permeabilisation/fixation buffers for intracellular staining were purchased from eBioscience (now Thermo Fisher Scientific). CRISPR/Cas9 reagents and TrueGuide synthetic single guide RNAs for AhR and HPRT1 positive control (AhR: CRISPR980382_SGM, HPRT: A35524) were obtained from Thermo Fisher and used as recommended by the manufacturer.

MACS Annexin V Microbead kit and MACS columns were purchased from Miltenyi Biotec (Bergisch Gladbach, Germany) and used according to manufacturer’s protocol. Cytokine analysis was performed using DuoSet ELISA kits by R&D Systems (Minneapolis, MN, USA).

### AhR knockout and selection protocol

Knockout procedure and the developed selection protocol are described in more detail in a previous publication (Sonnenburg et al. 2023). Briefly, THP-1 cells were seeded into 96-well round bottom plates at a density of 10,000 cells/well and incubated for 48 h in OptiMEM (Sigma) mixed with 400 ng TrueCut Cas9 protein v2, 2.4 pmol sgRNA, 0.9 µL Cas9 Plus reagent, and 0.3 µL CRISPRMAX Lipofectamine to a volume of 10 µL per well. Untreated cells incubated under the same conditions served as negative controls. Positive controls were treated with HPRT1 positive control sgRNA.

Edited cells were transferred to RPMI and passaged several times before FACS analysis using intracellular staining with anti-AhR-PE (eBioscience). Editing success in HPRT1 positive controls was confirmed by incubation of cells with 6-TG, which induces cell death in unedited cells. Survival in edited cells after 6-TG treatment was compared to that of unedited controls.

To enrich AhR-deficient cells after knockout, we have established a selection protocol. DBP was shown to induce apoptosis in mouse neuronal cells mediated by AhR (Wojtowicz et al. 2017). We used this principle to induce apoptosis in unedited cells. After incubation with DBP, apoptotic cells were removed from the culture by MACS magnetic sorting using Annexin V beads as described in the manufacturer’s protocol (Miltenyi Biotech GmbH). This procedure was repeated several times to maintain high levels of AhR-k.o. cells and directly before using cells in the eLCSA.

### eLCSA tier 1: HaCaT coculture

HaCaT were seeded onto 12-well tissue culture plates at a density of 2x10^5^ cells/well in DMEM supplemented with 10% FCS and incubated overnight at standard conditions. On day 1 of coculture, AhR-k.o. THP-1 were removed from selection medium and sorted using Annexin V beads. For PD-L1 blocking, cells were treated with 1 µg/mL anti-PD-L1 (Thermo Fisher, clone: MIH1) and incubated for one hour. Isotype control cells were treated equally with Mouse IgG1 kappa isotype control (Thermo Fisher, clone: P3.6.2.8.1). Supernatant was aspirated from wells with adherent HaCaT cells. Wild type (w.t.) THP-1 and sorted AhR-k.o. THP-1 with or without PD-L1 blockade were seeded onto HaCaT in 2 mL RPMI-medium at a density of 1x10^6^ cells/well. Test substances were added in duplicates or triplicates in concentrations previously determined to activate THP-1 but to avoid cytotoxicity: 10 µmol/L DNCB, 200 µmol/L MBT, and 150 µmol/L SLS. Untreated cells served as controls.

After another 48 h of incubation, aliquots of floating cells were taken for flow cytometric analysis of CD86 and CD54 expression, and viability. Cells were stained with anti-CD86-PE (eBioscience, clone: IT2.2), anti-CD54-FITC (Invitrogen, clone: RR1/1), and 7-AAD viability solution. Controls consisted of corresponding isotype controls and cells stained with anti-PD-L1-PE (eBioscience, clone: MIH1) or intracellularly with anti-AhR-PE (Invitrogen, clone: FF3399) to determine baseline levels of said receptors in blocked and genetically modified populations after the incubation period.

CD86 and CD54 expressions on modified and test substance-treated cells were compared to expressions on untreated w.t. controls. Therefore, results were corrected for the modification effect by dividing mean fluorescence intensity (MFI) data for untreated modified controls by MFI data for untreated w.t. controls and subtracting this expression change from relative values for treated modified cells as compared to w.t. controls.

At least three independent experiments were performed. Replicates yielding highest CD86 and CD54 expressions and lowest viability losses as compared to untreated controls were chosen for Jurkat coculture (tier 2).

### eLCSA tier 2: Jurkat coculture

In 96-well round bottom plates (Thermo Fisher), 2.5x10^4^ Jurkat cells in RPMI-1640 supplemented with 10% FCS and 2 mmol/L L-Glutamine (Merck) were added to each well. Cell numbers in chosen tier 1 replicates were determined and medium volumes to yield 5000 cells were calculated. These volumes were added to Jurkat cells in quadruples. Media volumes from HaCaT coculture (tier 1) added to Jurkat coculture did not exceed 11% of the final tier 2 volume per well. Controls consisted of 2.5x10^4^ Jurkat cells/well alone as negative controls and Jurkat treated with 10 µL CD3/CD28 microbeads (Dynabeads Human T-Activator, Invitrogen) as proliferation controls. Last, wells were filled up to 250 µL with RPMI. After 5 days of incubation at standard conditions, cells were harvested and prepared for FACS analysis. Supernatants were collected, pooled from quadruples, and frozen at −20 °C until cytokine analysis. Cells were stained with anti-CD3-PE (eBioscience) and intracellularly with anti-Ki-67-FITC (clone: SolA15, eBioscience). Proliferation status was measured by Ki-67 incorporation and a gate was set based on proliferation in Jurkat control cultures treated with anti-CD3/CD28 beads to determine the fraction of highly proliferating cells. Baseline effect was determined by comparison of CD3 expression on Jurkat in culture with untreated THP-1 modifications (controls from tier 1) with CD3 expression on Jurkat in monoculture.

Cytokine analysis of supernatants included MIP-1beta, MIP-3alpha, IL-8, and TNF-alpha. Cytokine concentrations in Jurkat coculture medium were corrected for respective added medium volumes from HaCaT cocultures. Relative cytokine concentrations were calculated by comparison of cocultures with untreated THP-1 modifications to cocultures containing treated THP-1 of the same modification.

### Software and statistical methods

Flow cytometric measurements were analysed using WinList 3D 8.0 (Verity Software House, Topsham, ME, USA). All graphical analyses were created using GraphPad Prism 8 (GraphPad Software, San Diego, CA, USA).

Single experimental values were calculated from raw data as described in the sections above. Sets of single values were analysed for outliers using the respective functionality in GraphPad Prism (settings: ROUT method, *Q* = 0.1%). Identified outliers were excluded from further analysis. Unpaired t-tests were used to compare two groups. Welch correction was employed in case of differing standard deviations (SD) between groups. For comparison of more than two groups, one-way ANOVA and Dunnett’s Test for post hoc analysis were used. Relative marker expression or concentration values were compared to a normalised control by one-sample *t* test. Nomenclature for significance was applied according to New England Journal of Medicine standards. All calculations were computed with the statistics tools provided by GraphPad Prism, version 8.

## Results

### eLCSA tier 1: HaCaT coculture

Results for AhR knockout experiments and establishment of selection protocol are described in more detail elsewhere (Sonnenburg et al. 2023). In summary, CRISPR/Cas9 editing and selection protocol resulted in a significantly lower percentage of AhR-positive THP-1 cells (median of 33%) in treated samples as compared to unedited controls. Blockade of PD-L1 with a monoclonal antibody resulted in statistically significantly reduced percentages of PD-L1-positive cells (mean of ~ 5%) in w.t. THP-1 populations and AhR-k.o. populations.

**Fig. 1:**
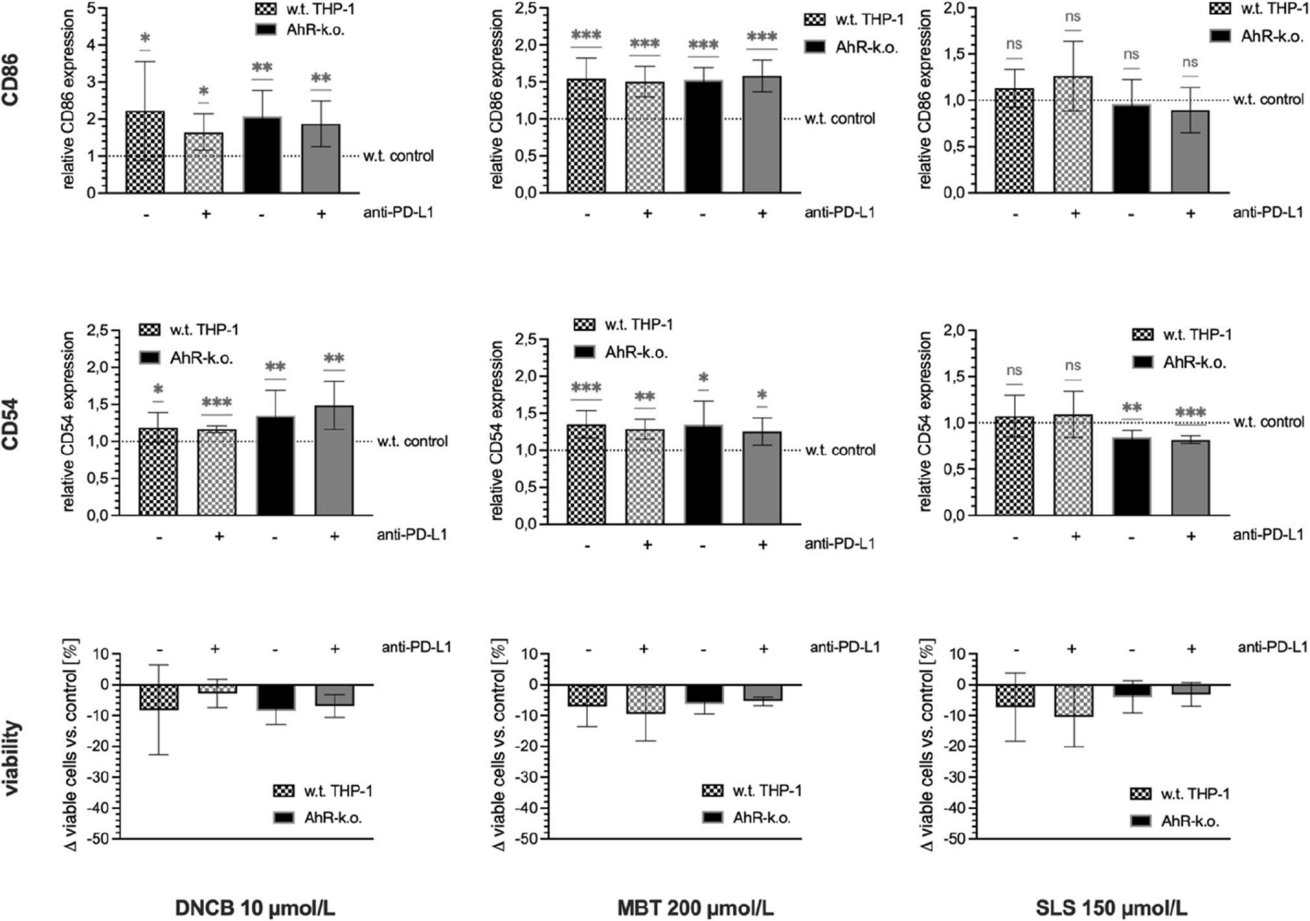
Relative expressions of CD86 (top) and CD54 (middle) on THP-1 modifications after treatment with 10 µmol/L DNCB (left), 200 µmol/L MBT (middle), or 150 µmol/L SLS (right) as compared to untreated wild type controls after two days of coculture with HaCaT keratinocytes. Bottom: Change in viability of treated cells as compared to respective controls in coculture with HaCaT. Means ± SD from three to five independent experiments

For treatment with test substances in the new series of experiments, we chose test substance concentrations that did not induce pronounced cytotoxicity as measured by viability staining with 7-AAD (Fig. 1). When treated with sensitisers in coculture with HaCaT keratinocytes, THP-1 upregulated CD86 and CD54 as compared to untreated wild type controls. Upregulation of CD86 expression was at similar level on all THP-1 modifications after treatment with 10 µmol/L DNCB or 200 µmol/L MBT. Interestingly, for CD54, upregulation was enhanced in AhR-k.o. THP-1 treated with DNCB and further increased by anti-PD-L1. In line with these observations, treatment with a non-cytotoxic concentration of irritant SLS led to a slight, although not statistically significant, upregulation of CD86 and CD54 on w.t. THP-1, whereas SLS treatment of AhR-k.o. THP-1 caused a downregulation of both markers on these cells. For CD54 this downregulation was statistically significant.

**Fig. 2:**
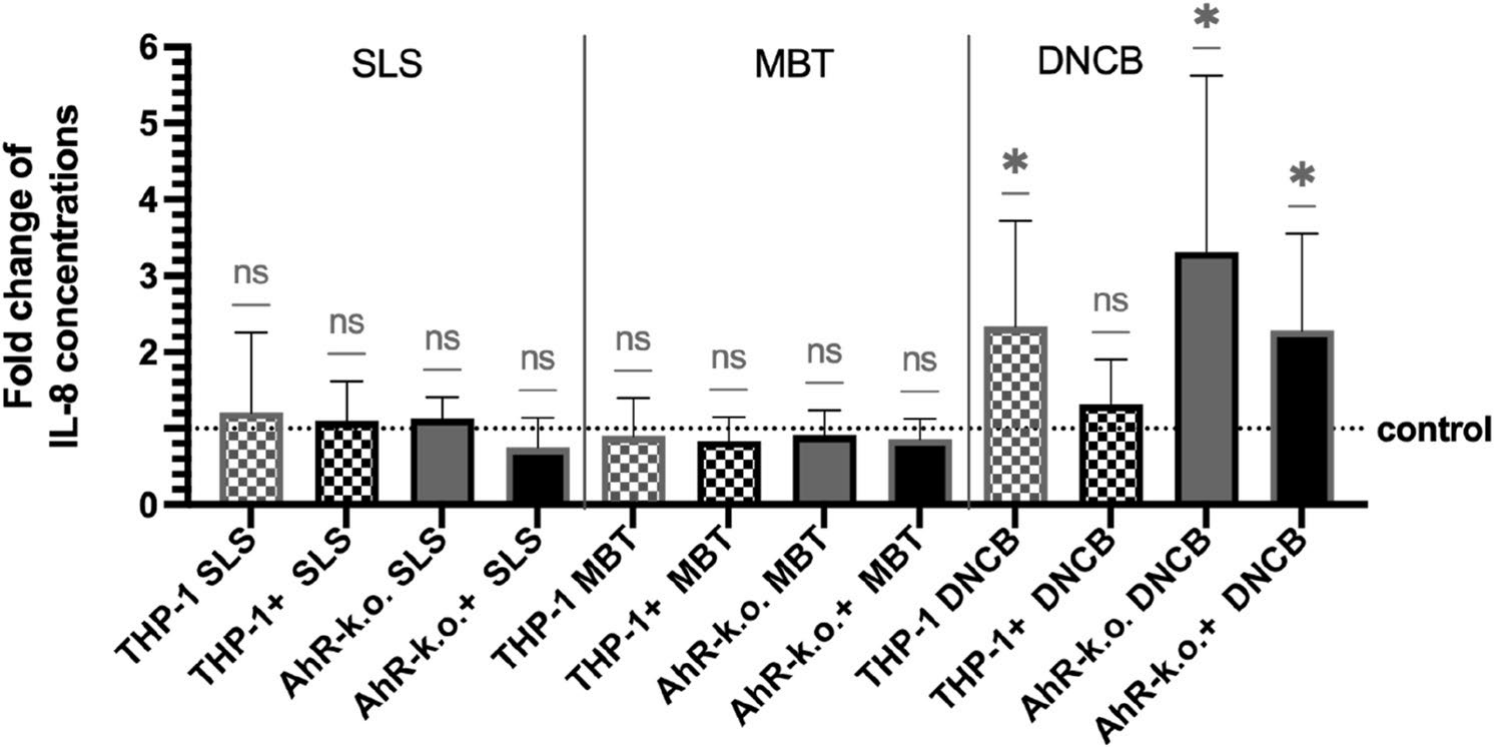
Relative values for IL-8 concentrations in supernatants of tier 1: HaCaT coculture with w.t. THP-1 (chequered bars) or AhR-k.o. THP-1 (plain bars) without (grey) or with (black) PD-L1 blockade, respectively, as compared to untreated controls of the same modification. THP-1: wild type cells, AhR-k.o.: AhR-knockout THP-1, + : cells treated with anti-PD-L1, SLS: treated with 150 µmol/L SLS, MBT: treated with 200 µmol/L MBT, DNCB: treated with 10 µmol/L DNCB. N = 4, means ± SD

As a first step to incorporate a read-out for keratinocyte activation (KE2), IL-8 was measured by ELISA in supernatants of tier 1 samples. When compared to controls of the same modification (wild type, wild type with blocked PD-L1, AhR-k.o., AhR-k.o. with blocked PD-L1), DNCB treatment led to a significant IL-8 concentration change in AhR-k.o. cultures with and without PD-L1 blockade and in w.t. THP-1 (2.3-fold, 3.3-fold, and 2.3-fold, respectively; Fig. 2). Relative secretion of IL-8 after SLS treatment slightly increased in cultures with w.t. THP-1 with and without PD-L1 blockade and AhR-k.o. cocultures (ranging from 1.1-fold in AhR-k.o. and PD-L1 blocked w.t. THP-1 cultures to 1.2-fold in w.t. THP-1 cultures). In contrast, cocultures of HaCaT keratinocytes and PD-L1 blocked AhR-k.o. cells that were treated with SLS secreted less IL-8 than their respective control cultures (0.75-fold). A consistent but non-significant and slight decrease of relative IL-8 concentrations was measured in cultures treated with MBT (0.9–0.8-fold). As a result, in the absence of T cells, release of IL-8 after treatment with a strong allergen was enhanced by AhR-knockout of THP-1 cells.

### eLCSA tier 2: Jurkat coculture

**Fig. 3:**
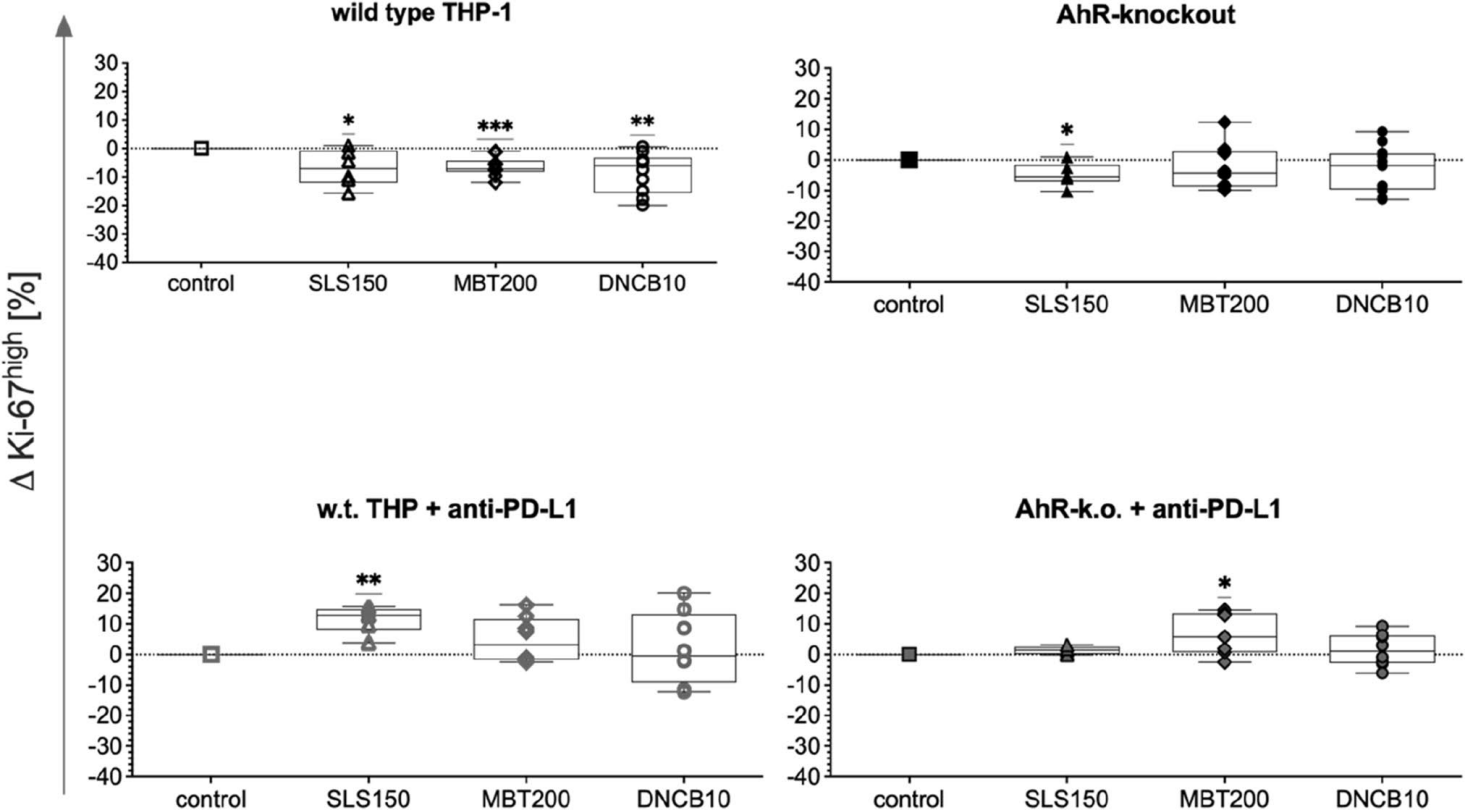
Changes in percentages of highly proliferating CD3^+^ cells after five days in cocultures of Jurkat T cells with THP-1 modifications in tier 2 after prior treatment of THP-1 with test substances (150 µmol/L SLS [triangles], 200 µmol/L MBT [diamonds], 10 µmol/L DNCB [circles]) in coculture with HaCaT (tier 1) as measured by anti-Ki-67 incorporation and compared to untreated cocultures of the same modification (squares). Single values, ranges, and medians from five independent experiments

After coculture with test substance-treated THP-1, lower percentages of highly proliferating Jurkat (Ki-67^high^) were detected in treated compared to untreated cocultures with w.t. and AhR-k.o THP-1 (Fig. 3). In contrast, if additionally blocked for PD-L1, w.t. and AhR-k.o. THP-1 treated with SLS or MBT, respectively, induced proliferation of cocultivated Jurkat as compared to untreated cells of the same modification.

**Fig. 4:**
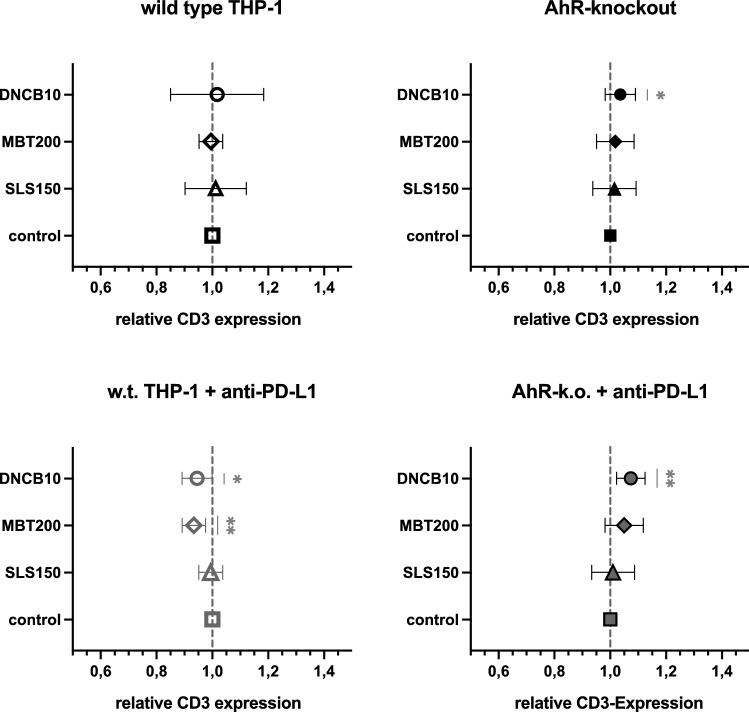
Relative CD3 expression in highly proliferating cells after five days in cocultures of Jurkat with THP-1 modifications treated with test substances (150 µmol/L SLS—triangles, 200 µmol/L MBT—diamonds, and 10 µmol/L DNCB—circles) in prior HaCaT coculture as compared to untreated control cocultures (control—squares). Means ± SD from three to five independent experiments

No alteration of CD3 expression on highly proliferating cocultivated Jurkat T cells was induced in w.t. THP-1 after stimulation with any of the test substances when compared to cocultures with untreated controls (Fig. 4). As the most interesting finding, DNCB-treated AhR-k.o. THP-1 with or without PD-L1 blockade induced a statistically significant increase in CD3 expression on cocultivated Jurkat. An induction was also measured in cocultures with MBT-treated AhR-k.o. cells with blocked PD-L1. PD-L1 blockade alone led to a statistically significantly reduced CD3 expression on Jurkat cocultivated with sensitiser-treated THP-1. Thus, AhR-knockout and blocking of PD-L1 had an over-additive effect on the induction of CD3 on highly proliferating cocultured Jurkat. Of note, in none of the cocultures with SLS treated THP-1 modifications CD3 expression on highly proliferating Jurkat was altered.

**Fig. 5:**
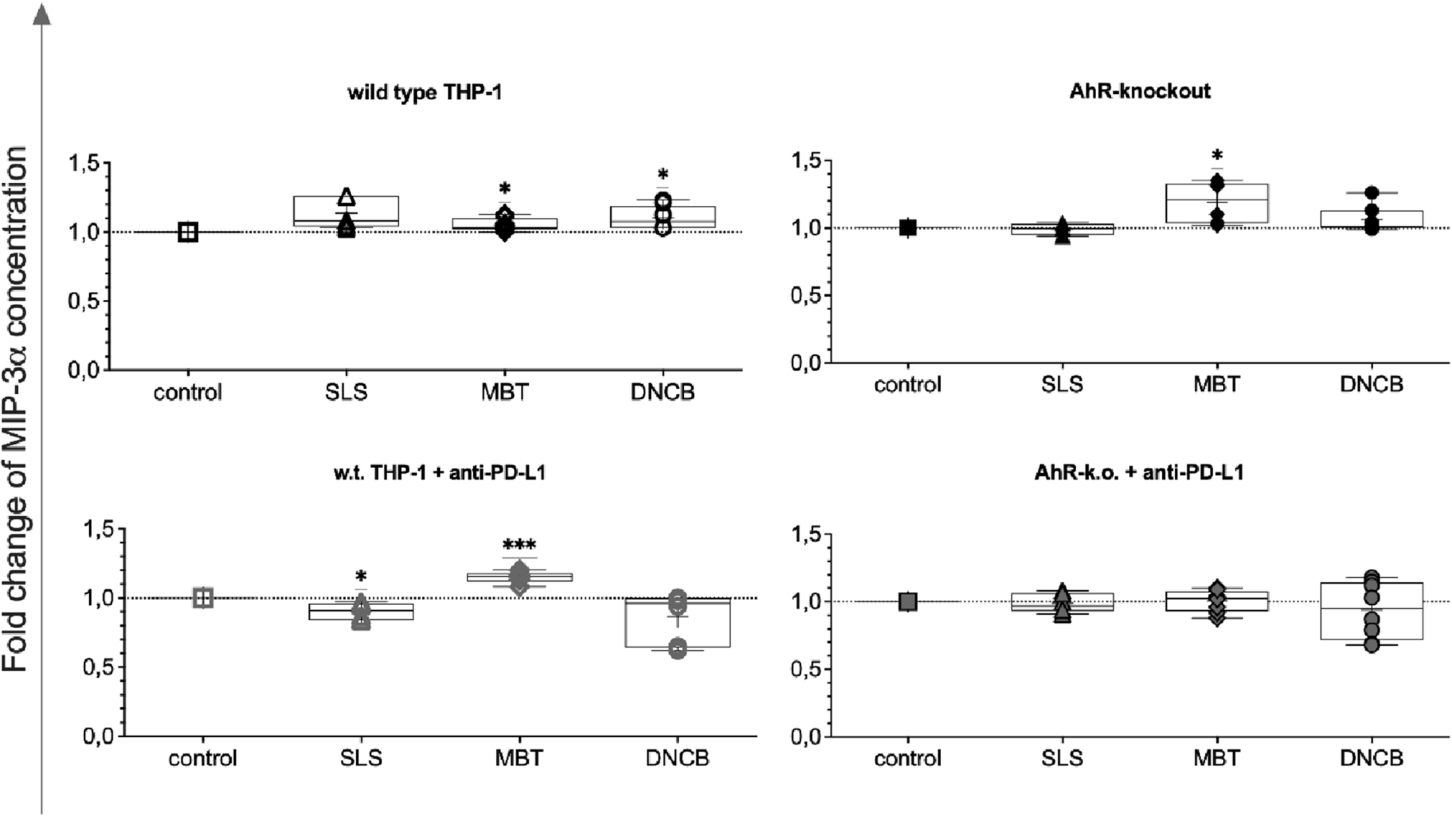
Relative concentrations of MIP-3α in cocultures of Jurkat T cells with test substance-treated THP-1 modifications (150 µmol/L SLS [triangles], 200 µmol/L MBT [diamonds], 10 µmol/L DNCB [circles]) after five days as compared to cocultures with untreated monocytes of the same modification. Single values, ranges, and medians from three to four independent experiments

In our experiments, four cytokines were identified in coculture supernatants of the eLCSA: MIP-3α, MIP-1β, TNF-α, and IL-8. Slight increases in MIP-3α concentrations as compared to control cocultures were detected in test substance-treated w.t. THP-1 cocultures (Fig. 5, top left). Treatment of single AhR-k.o. cells or PD-L1-blocked THP-1 with MBT lead to a statistically significant increase in MIP-3α concentrations in the cocultures as compared to the respective controls, combinatory block and knockout, or compared to irritant stimulation by SLS.

**Fig. 6:**
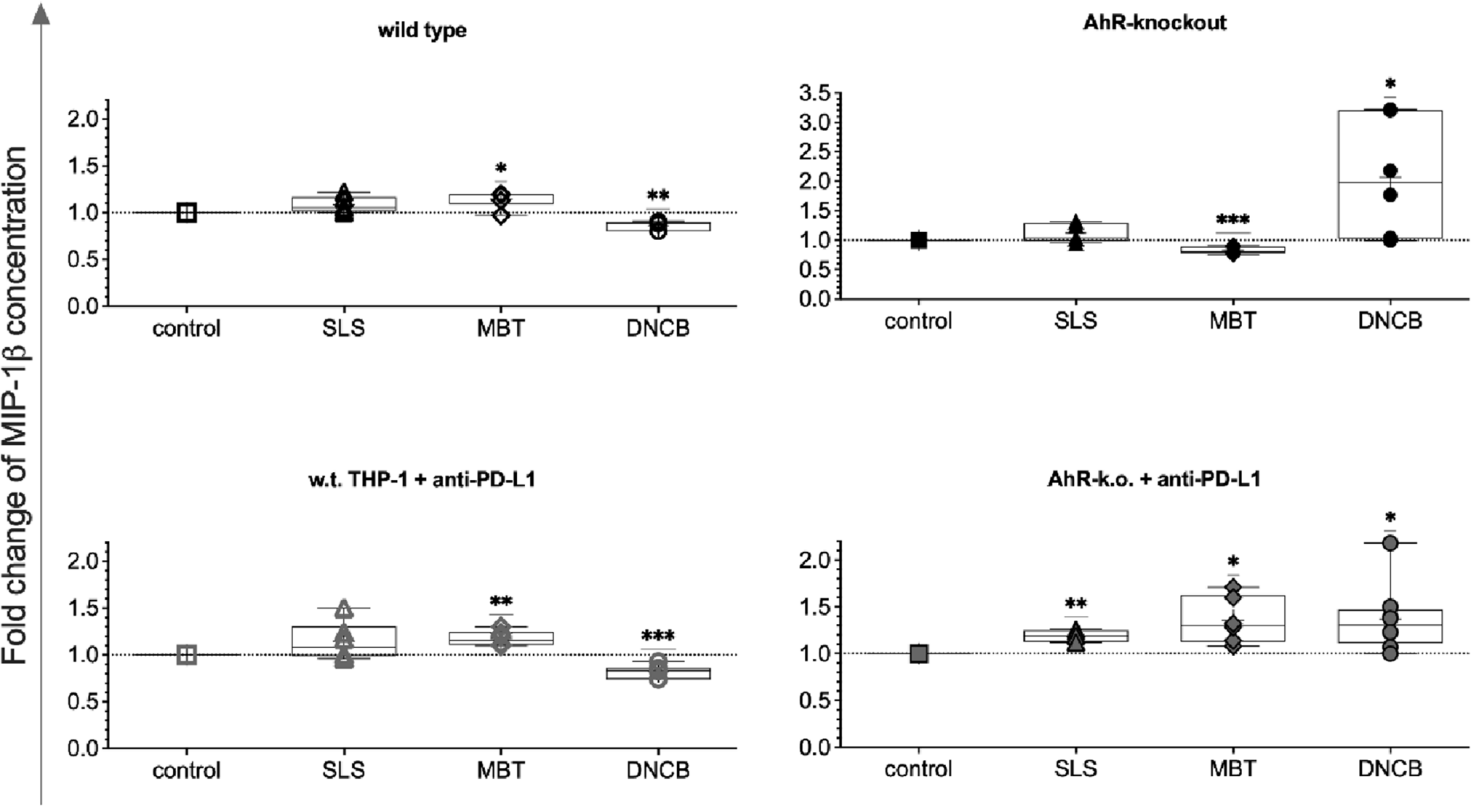
Relative concentrations of MIP-1β in cocultures of Jurkat T cells with test substance-treated THP-1 modifications (150 µmol/L SLS [triangles], 200 µmol/L MBT [diamonds], 10 µmol/L DNCB [circles]) after five days as compared to cocultures with untreated THP-1 of the same modification. Single values, ranges, and medians from three to four independent experiments

In cocultures of Jurkat with test substance-treated PD-L1 blocked AhR-k.o. THP-1, MIP-1β concentrations were statistically significantly higher than in control cocultures with untreated cells of the same modification and also higher than in cells stimulated by SLS (Fig. 6, bottom right). For DNCB, AhR knockout alone enhanced release level of MIP-1β, thus allowing for a clearer distinction between sensitisers and irritant.

**Fig. 7:**
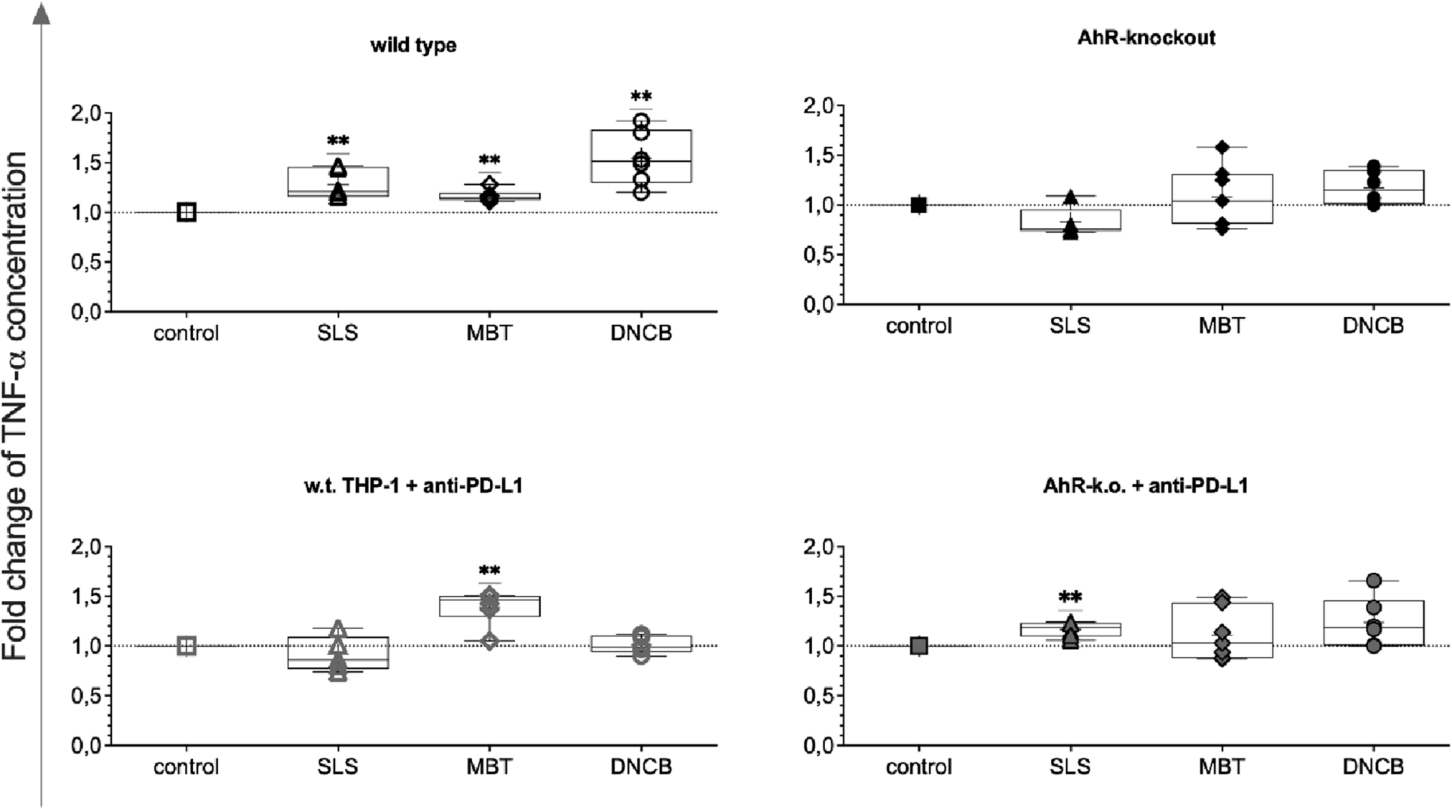
Relative concentrations of TNF-α in cocultures of Jurkat T cells with test substance-treated THP-1 modifications (150 µmol/L SLS [triangles], 200 µmol/L MBT [diamonds], 10 µmol/L DNCB [circles]) after five days as compared to cocultures with untreated monocytes of the same modification. Single values, ranges, and medians from three to four independent experiments

As seen for MIP-3α, TNF-α concentrations in cocultures with test substance-treated w.t. THP-1 were increased above control levels for all test substances (Fig. 7, top left). To a lesser extent, this was also true for cocultures with PD-L1 blocked AhR-k.o. cells (Fig. 7, bottom right). Opposite effects were observed for cocultures with MBT and DNCB-treated PD-L1 blocked THP-1 and AhR-k.o. THP-1. Thus, increased TNF-α concentrations were measured in cocultures with MBT-treated PD-L1 blocked THP-1 and DNCB-treated AhR-k.o. cells.

**Fig. 8:**
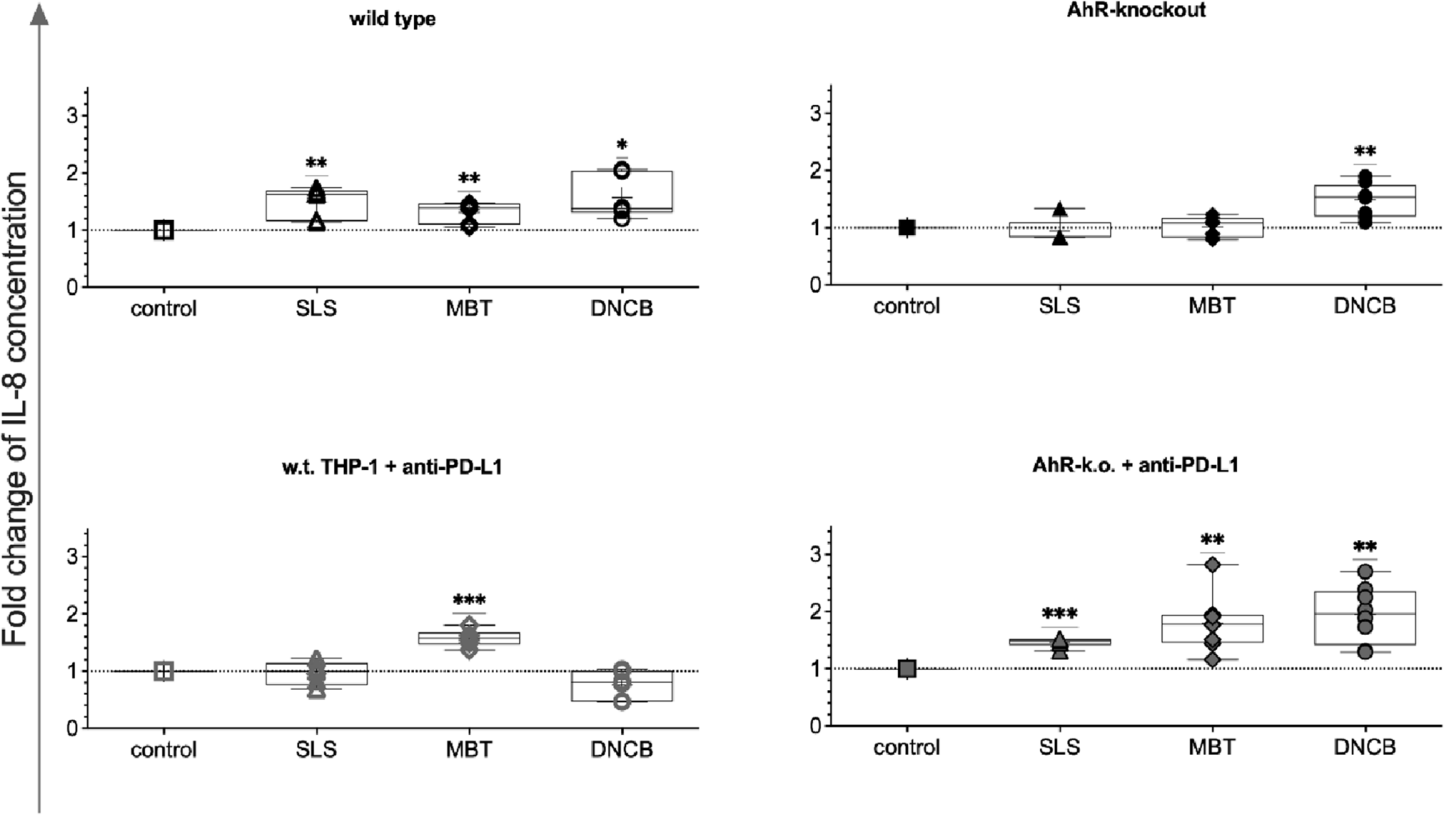
Relative concentrations of IL-8 in cocultures of Jurkat T cells with test substance-treated THP-1 modifications (150 µmol/L SLS [triangles], 200 µmol/L MBT [diamonds], 10 µmol/L DNCB [circles]) after five days as compared to cocultures with untreated monocytes of the same modification. Single values, ranges, and medians from three to four independent experiments. Note: Although IL-8 was also detected in media of tier 1, only insignificant amounts were transferred to tier 2 with THP-1 modifications (1.7% of the concentrations measured in tier 2 at most, data not shown)

Cocultures with substance-treated w.t. THP-1 or PD-L1 blocked AhR-k.o. cells all contained higher levels of IL-8 than respective untreated control cocultures (Fig. 8). When PD-L1 was blocked on w.t. THP-1, SLS treatment failed to alter IL-8 concentrations in subsequent Jurkat cocultures, while prior MBT treatment led to elevated IL-8 concentrations as compared to untreated control cocultures. Cocultures with AhR-k.o. cells after prior treatment with DNCB but not SLS led to a statistically significant increase in IL-8 concentrations.

In summary, PD-L1 blockade or AhR knockout alone advanced subsequent Jurkat response by release of allergen-specific cytokines and immunomarkers, thereby facilitating discrimination between irritant and sensitisers. However, when both modifications were applied, combinatory effects were found specifically on CD54 and CD3 expression and IL-8 release.

Therefore, combined results from tier 1 and 2 indicate that using THP-1 with AhR knockout alone and in combination with PD-L1 blockade as well as PD-L1 blockade alone leads to a more distinctive pattern of marker responses based on sensitiser potency. For graphical appraisal of the overall results see supplemental figures.

## Discussion

### Breaking of inhibitory signals in immune cell-based sensitisation assays

To overcome the immune-inhibitory signals interfering between different cells types of our eLCSA, the molecular approach of genome editing was used in this study. As dendritic cells interact with both keratinocytes and T cells which are mechanistically involved in the pathogenesis of skin sensitisation (Peiser et al. 2012), the expression of immune-inhibitory molecules PD-L1 and AhR was experimentally blocked and altered on dendritic cell-similar THP-1. A final combination of CRISPR/Cas9 editing, dibutylphthalate treatment, and magnetic sorting achieved one third less AhR-positive THP-1 cells. Anti-PD-L1 monoclonal antibody was demonstrated to efficiently block binding sites of the receptor in THP-1 (Sonnenburg et al. 2023). Up to now, to our knowledge no reports exist on knockout of immunoinhibitory molecules such as AhR or B7H molecules in human cells used in immunoassays. However, in AhR^+/−^ mice lacking exon 2 on a C57BL/6 background, a loss of AhR induced decreased expression of anti-inflammatory cytokine IL-10 in LPS-induced macrophages, thereby providing further evidence on the physiological role of AhR in regulating the inflammatory response (Zhu et al. 2018). This fits well with our data provided here that vice versa AhR knockout may enhance inflammatory response induced by specific stimuli such as skin sensitisers.

### Tier 1: modulation of dendritic cell markers enhanced by AhR-k.o. and anti-PD-L1

As hypothesised, in this study AhR-k.o. combined with anti-PD-L1 strongly increased the response of THP-1 to the allergen DNCB as shown for the marker CD54 (Fig. 1). In contrast, treatment of AhR-k.o. THP-1 with or without additional PD-L1 blockade with the irritant SLS significantly reduced levels of CD54 after coculture with HaCaT keratinocytes. Expression of CD86 was also slightly reduced in these cells after SLS treatment. Since CD54 acts as adhesion molecule for the binding of T cells, reduced CD54 expression on irritant stimulated AhR-k.o. THP-1 should hamper subsequent T cell activation in our test system.

These results could be regarded as a proof-of-principle for enhancing the level of response to allergens by modulating inhibitory molecules. Thus, AhR-k.o. and anti-PD-L1 could also be proposed to enhance sensitivity in different regulatory sensitisation assays including OECD endorsed h-CLAT. In a mouse model, AhR expression in C3H/HeOuJ mice was triggered by treatment with TCDD followed by sensitisation to peanut extract (Schulz et al. 2013). While the absolute number of dendritic cells nearly doubled, expression of CD54 was not increased. This is in accordance with our results showing increase CD54 after allergen exposure if not AhR stimulation but knockout was applied.

### Tier 1: AhR-knockout and PD-L1 blockade allow discrimination of sensitisers from irritant by IL-8 secretion of THP-1

The pro-inflammatory cytokine IL-8 is produced by epithelial cells like keratinocytes as well as by monocytes (Baggiolini and Clark-Lewis 1992). Our results for IL-8 secretion into culture media of tier 1 indicate that secretion of IL-8 by THP-1 is regulated by AhR and PD-L1 (Fig. 2).

Involvement of AhR in IL-8 regulation was previously shown by another working group in the myeloid cell line KBM-7 and primary monocyte-derived macrophages (Zablocki-Thomas et al. 2020). Our experiments show that blockade of PD-L1 additionally reduces IL-8 production by AhR-k.o. THP-1. Interestingly, stimulation with a strong allergen like DNCB overcame inhibition of IL-8 production as evidenced by similar concentrations in respective cocultures as compared to w.t. THP-1 cocultures. Therefore, relative induction of IL-8 was higher in knockout cocultures than in w.t. cocultures leading to a wider dynamic range for this parameter. However, MBT failed to break AhR-knockout and PD-L1 blockade mediated inhibition of IL-8. In contrast, in cocultures with PD-L1 blocked AhR-k.o. cells stimulated with SLS relative IL-8 concentrations were even lower as compared to respective control cocultures (although not statistically significant). This again may contribute to a clearer distinction between allergens and irritants in the eLCSA compared with conventional methods. However, further investigations including detection of other keratinocyte activation markers are needed to fully introduce a read-out for KE1 in the eLCSA.

### Tier 2: AhR-k.o. and anti-PD-L1 enhance sensitiser response of CD3

Previous work in our working group showed the functional integration of T cells as immune effectors into a cell-based sensitisation assay (Frombach et al. 2018). Here, we propose CD3 on Jurkat cells as an additional predictive marker. As indispensable part of the functional T cell receptor (TCR) complex, CD3 is needed for signal transduction by phosphorylation of cytoplasmatic ITAM regions upon binding of TCR (Dong et al. 2019). In cocultured Jurkat cells, DNCB-treated AhR-k.o. THP-1 increased the level of CD3 in the eLCSA as compared to cocultures with untreated controls (Fig. 4). This effect was even more pronounced in cocultures with additional PD-L1 blockade. Moreover, Jurkat in coculture with MBT-treated PD-L1-blocked AhR-k.o. THP-1 also increased CD3 expression. This increase was not observed for irritant SLS in any coculture and not at all in cocultures with w.t. THP-1. In contrast, prior treatment with sensitisers of only PD-L1 blocked THP-1 led to significantly reduced CD3 expression in cocultivated Jurkat T cells. Thus, combination of PD-L1 blockade and AhR-knockout in THP-1 acted over-additive on the CD3 expression of cocultivated Jurkat.

This seemingly paradox results may be explained by the concurrent action of different mechanisms. We have previously shown that AhR-knockout induces CD86 and CD54 expression on unstimulated THP-1 while PD-L1 blockade does neither induce nor alter this effect (Sonnenburg et al. 2023). PD-L1 was shown to *cis*-dimerise on antigen-presenting cells with CD80, another dendritic cell marker that acts similar to CD86, i.e. activates T cells, while dimerisation with PD-L1 inhibits PD-L1/PD-1 interaction (Sugiura et al. 2019). Considering parallel down-regulation of B7 molecules CD86 and CD80 in AhR-activated moDC (Wang et al. 2014), it is reasonable to assume that CD80 is also upregulated in AhR-knockout THP-1 [as was shown for CD86 in our previous study, (Sonnenburg et al. 2023)] enhancing dimerisation with PD-L1. Thus, several activating mechanisms would be enhanced in AhR-k.o. cells with additionally blocked PD-L1. In cells only blocked for PD-L1 expression of CD80 and CD86 would be significantly lower but still elevated by sensitiser stimulus leading to activation of other inhibitory mechanisms in activated T cells such as expression of CTLA4. This receptor collocates with CD28, which is the binding partner of CD86 and CD80, and exerts regulatory action (Linsley et al. 1992). Since only around 10% of the cocultuture’s medium still contained test substance after transferral of treated THP-1 to Jurkat culture, it is safe to assume that Jurkat stimulation was by direct signal transduction from activated THP-1 rather than contact with test substances.

In conclusion, for a possible read-out both modifications (PD-L1 blockade alone or in combination with AhR-knockout) proved useful to induce a measurable sensitiser-mediated alteration of a T cell marker.

### AhR-k.o. and anti-PD-L1 increase sensitiser-induced response of MIP-3α, MIP-1β, TNF-α, and IL-8

Among a panel of 12 cytokines analysed (not shown), MIP-3α, MIP-1β, TNF-α, and IL-8 were found to be affected in the supernatant of eLCSA by the two modifications of THP-1 cells. In general, in this study the identification of the source of the soluble factors, that could be keratinocytes, THP-1 or T cells, was not a primary goal. However, for potency derivation and further regulatory purposes rather the kinetics and range of release than the definition of the source might be relevant.

In comparison to cocultures with w.t. THP-1, MIP-3α concentrations were elevated if THP-1 were treated with MBT and when the AhR-k.o. system was used (Fig. 5). In contrast, previous SLS treatment of AhR-k.o. THP-1 led to a reduction of MIP-3α production down to control levels. In addition, DNCB treatment induced MIP-3α secretion. MIP-3α (CCL20) is known to be involved in pathogenesis of allergies of the upper respiratory tract (Ahrens et al. 2015; Post et al. 2015) and in steady state migration of dendritic cells in the skin (Charbonnier et al. 1999). We propose MIP-3α as an additional allergy marker for the application in skin sensitisation assays.

Regulation of a further inflammatory chemokine, MIP-1β (CCL4), was observed in response to MBT and DNCB and was enhanced in cocultures with PD-L1-blocked THP-1 (Fig. 6). MIP-1β was detected on mRNA and protein level after stimulation by DNCB or MBT by another working group (Hirota and Moro 2006). In a further study with THP-1 cells, all sensitising chemicals tested, including DNCB, induced release of MIP-1β and non-sensitizers with one exception were truly negative (Lim et al. 2008). In a sensitisation assay of MUTZ-3-derived Langerhans cells, HaCaT and primary dermal fibroblasts, MIP-1 β was used in combination with further biomarkers GM-CSF and IL-8 to improve the detection of pre- and pro-haptens (Lee et al. 2018). However, until now application of blocking for immunoregulatory molecules and its effect on MIP-1β were not reported.

TNF-α is released at the early stage of sensitisation and also in the effector phase after allergen re-exposure by sensitised epithelial barriers and dendritic cells. (Ahmad et al. 2018). For TNF-α, in our study blocking of PD-L1 increased response to MBT (Fig. 7). Only few studies investigated the effect of PD-L1 blocking on immune cells. Two recent reports found enhanced release of TNF-α in macrophages and moDCs (Bar et al. 2020; Lu et al. 2019).

Endogenous ligands for activation of AhR, FICZ or ITE, were shown to inhibit the production TNF-α in moDCs (Wang et al. 2014) which may indicate a regulatory role of AhR for TNF-α. This is supported by our previous findings where cocultures with AhR-k.o. THP-1 contained higher levels of TNF-α than respective w.t. cocultures (Sonnenburg et al. 2023). The stronger the sensitising potency of the test substance, the higher were the measured relative TNF-α concentrations in cocultures with AhR-k.o. THP-1. Hence, SLS treatment reduced TNF-α concentrations as compared to untreated controls but DNCB treatment induced higher TNF-α concentrations in the cocultures of tier 2.

A role of TNF-α in sensitisation in vivo and in vitro was previously shown (Ahmad et al. 2018) and our data indicate that modification of monocytic cells such as THP-1 helps to enhance distinction between allergen and an irritant. Thus, a combination of detecting TNF-α in sensitisation assays and applying PD-L1 blocking and AhR knockout as proposed in this study is plausible.

In addition to CD86, IL-8 was established to be the best validated marker for in vitro sensitisation assays using dendritic cells or dendritic cell-related cells such as THP-1 or MUTZ-3. For example, IL-8 is analysed in the reporter gene assay or IL-8 Luc assay as recommended by OECD TG442E (OECD 2018b). Therefore, IL-8 was used as an allergy marker with known predictivity to validate eLCSA. Detection of IL-8 was optimised in PD-L1 blocked and AhR-k.o. cocultures for DNCB and MBT, respectively, again providing much better distinction from vehicle controls and irritant SLS than it was seen in cocultures with w.t. THP-1 (Fig. 8).

## Conclusion

In conclusion, this study demonstrates for the first time that genome editing and modulation of immunoinhibitory molecules results in functional alteration of dendritic cells and T cells, still remaining sensitive and immunocompetent cells. This technical application may enhance read-out and allow detection of T cell responses in in vitro skin sensitisation assays. The eLCSA integrates all major players of the AOP on skin sensitisation (i.e. dendritic cells, keratinocytes, and T cells) in one single assay system. By application of AhR knockout in THP-1 together with antibody blockade of the PD-L1:PD-1 interaction we were able to increase the dynamic range of biomarker alteration induced by sensitising chemicals. This study serves as proof-of-principle evidence and supports further studies on the eLCSA with an extended set of test substances that are needed for verification and validation. This will be important to enhance predictivity and accuracy of immune cell based in vitro systems as compared with in vivo mouse and guinea pig assays.

## Supplementary Information

Below is the link to the electronic supplementary material.

Supplementary file1 (PDF 966 kb)

## Data Availability

The datasets generated and analysed during the current study are available from the corresponding author on reasonable request.
