# Supplementary material for: A new cell line based coculture system for skin sensitisation testing in one single assay using T cells, aryl hydrocarbon receptor knockout, and co-inhibitory blockage
Source: Arch Toxicol. 2023 May 5;97(6):1677–89. doi: 10.1007/s00204-023-03506-3 (PMC10182954; doi:10.1007/s00204-023-03506-3)
Supplement: Supplementary file 1 — Supplementary file1 (PDF 966 kb) [file 204_2023_3506_MOESM1_ESM.pdf]

Archives of Toxicology (2023)

"A new cell line based coculture system for skin sensitisation testing in one single assay using T cells, aryl hydrocarbon receptor knockout, and co-inhibitory blockage"

Sonnenburg A[1,2,3], Stahlmann R[1], Kreutz R[1], Peiser M[2,4]

[1] Institute for Clinical Pharmacology and Toxicology, Charité-Universitätsmedizin Berlin, Berlin, Germany

[2] Institute of Chemistry and Biochemistry, Freie Universität Berlin, Berlin, Germany

[3] Department Pesticides Safety, German Federal Institute for Risk Assessment, Berlin, Germany

[4] Department Food Safety, German Federal Institute for Risk Assessment, Berlin, Germany

Corresponding author: Anna Sonnenburg, anna.sonnenburg@bfr.bund.de

Supplemental Figures

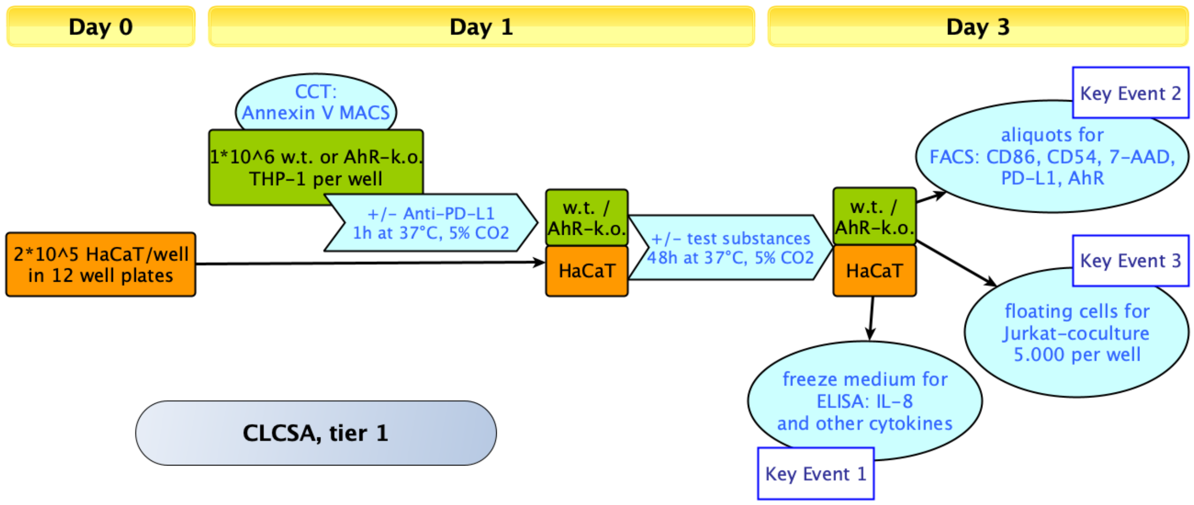

Figure 1. Schematic outline for tier 1 of the eLCSA – the HaCaT coculture with THP-1 modifications. Cells are seeded onto 12-well plates after a one hour incubation of monocytes with anti-PD-L1 or isotype control. Test substances are added in duplicates at appropriate concentrations. Following a 48-hour incubation period cells and supernatant are harvested. An aliquot of floating cells is analysed for dendritic cell markers to cover KE2. Five thousand cells each are used for subsequent tier 2 Jurkat coculture. Supernatant can be used to analyse keratinocyte activation for KE1.

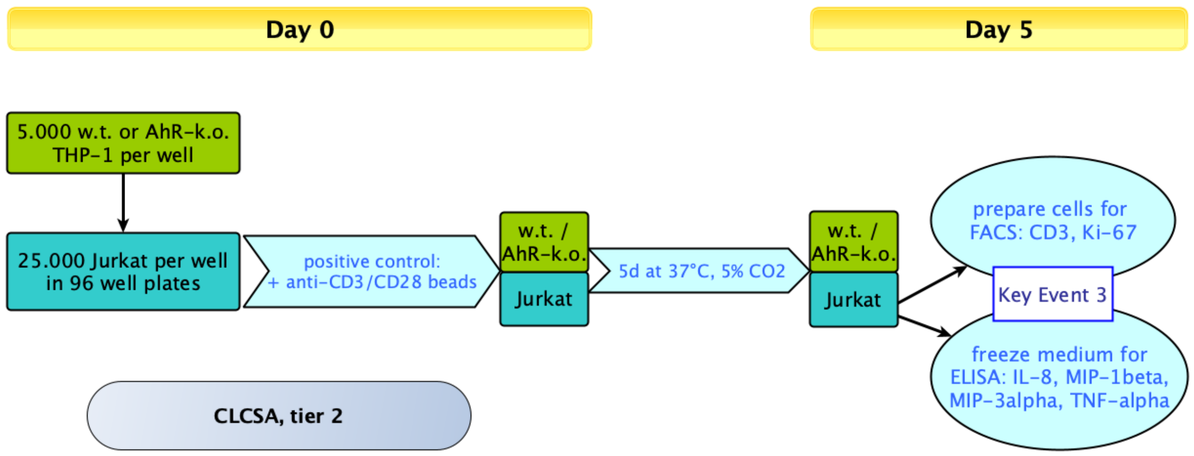

Figure 2. Schematic outline for tier 2 of the eLCSA – the Jurkat coculture with test substance treated THP-1 modifications from tier 1. THP-1 modifications and Jurkat T cells are seeded onto 96 well plates at a ratio of 1:5, controls consist of Jurkat in monoculture with or without anti-CD3/CD28 bead stimulation. After a five-day incubation period cells and supernatants are harvested for flow cytometric analysis of proliferation, CD3 expression, and ELISA to cover KE3.

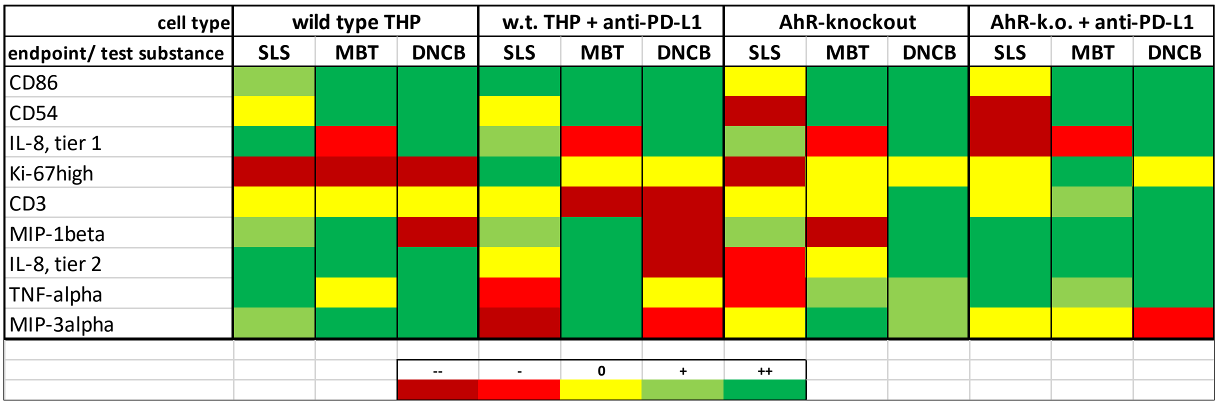

Figure 3. Heatmap comprising overall results from tier 1 and 2 of the eLCSA. Yellow: changes of less than 5 %, light red or green: 5 % ≤ change < 20 %, dark red or green: changes higher than 20 % or statistically significant. While PD-L1 blockade on w.t. THP-1 led to a colour shift to red for T cell parameters with increasing sensitisation potency of the test substance, AhR knockout led to a colour shift to green with increasing sensitisation potency of the test substance used.
